# Efficacy, safety and immunogenicity of etanercept biosimilars *versus* reference biologics in patients with rheumatoid arthritis: A meta-analysis

**DOI:** 10.3389/fphar.2023.1089272

**Published:** 2023-02-16

**Authors:** Rui Hu, Tao Yuan, Hui Wang, Jianglin Zhao, Liya Shi, Quankai Li, Chunmei Zhu, Na Su, Shengzhao Zhang

**Affiliations:** ^1^ Department of Pharmacy, Karamay Central Hospital, Karamay, China; ^2^ Department of Nephropathy and Rheumatology, Karamay Central Hospital, Karamay, China; ^3^ Department of Pharmacy, West China Hospital, Sichuan University, Chengdu, China; ^4^ West China School of Pharmacy, Sichuan University, Chengdu, China

**Keywords:** rheumatoid arthritis, etanercept, biosimilars, meta-analysis, reference biologic

## Abstract

**Background:** Although with the application of etanercept biosimilars in the field of rheumatoid arthritis, the evidences of their efficacy, safety, and immunogenicity are still limited. We conducted this meta-analysis to evaluate the efficacy, safety and immunogenicity of etanercept biosimilars for treating active rheumatoid arthritis compared to reference biologics (Enbrel^®^).

**Methods:** PubMed, Embase, Central, and ClinicalTrials.gov were searched for randomized controlled trials of etanercept biosimilars treated in adult patients diagnosed with rheumatoid arthritis from their earliest records to 15 August 2022. The outcomes included ACR20, ACR50, and ACR70 response rate at different time points from FAS or PPS, adverse events, and proportion of patients developed anti-drug antibodies. The risk of bias of each included study was assessed using the revised Cochrane Risk of Bias in Randomised Trials tool, and the certainty of evidence was rated according to the Grading of Recommendation Assessment, Development, and Evaluation.

**Results:** Six RCTs with 2432 patients were included in this meta-analysis. Etanercept biosimilars showed more benefits in ACR50 at 24 weeks from PPS [5 RCTs, OR = 1.22 (1.01, 1.47), *p* = 0.04, *I* ^*2*^ = 49%, high certainty], ACR50 at 1 year from PPS [3 RCTs, OR = 1.43 (1.10, 1.86), *p* < 0.01, *I* ^*2*^ = 0%, high certainty] or FAS [2 RCTs, OR = 1.36 (1.04, 1.78), *p* = 0.03, *I* ^*2*^ = 0%, high certainty], and ACR70 at 1 year from PPS [3 RCTs, OR = 1.32 (1.01, 1.71), *p* = 0.04, *I* ^*2*^ = 0%, high certainty]. In terms of other outcomes about efficacy, safety, and immunogenicity, the results showed that there was no significant difference between etanercept biosimilars and reference biologics, and the certainty of evidences ranged from low to moderate.

**Conclusion:** Etanercept biosimilars showed more benefits in ACR50 response rate at 1 year than reference biologics (Enbrel^®^), other outcomes for clinical efficacy, safety, and immunogenicity of etanercept biosimilars were comparable with originator in patients with rheumatoid arthritis.

**Systematic Review Registration:** PROSPERO, identifier CRD42022358709

## 1 Introduction

Rheumatoid arthritis (RA) is a type of autoimmune inflammatory arthritis with joint pain, stiffness, swelling, as well as systemic manifestations (Smolen et al., 2016). According to relevant epidemiological data, RA showed a global prevalence of 0.22% (Abbafati et al., 2020). RA not only can lead to progressive joint damages, but also may lead to the destruction of cartilage and bone, reducing patients’ quality of life and even disability, if treatment is delayed or not controlled properly (Laugisch et al., 2016; Cush, 2021).

General management measures for RA include disease-modifying anti-rheumatic drugs (DMARDs), anti-inflammatory therapy with non-steroidal anti-inflammatory drugs (NSAIDs), glucocorticoids or biologicals (Allen et al., 2018; Smolen et al., 2020). However, many of these drugs become less effective and exhibit increased toxicity over time (Kerschbaumer et al., 2020). Etanercept as a biological disease-modifying anti-rheumatic drug (bDMARD) has been shown to have salutary treatment of moderate-to-severe rheumatoid arthritis (RA) psoriatic arthritis, axial spondyloarthritis and psoriasis in adults, and juvenile idiopathic arthritis in pediatric patients (Goldenberg, 1999; Genovese et al., 2002). In addition, it has been reported that etanercept leads to less serious adverse reactions when compared with the traditional or conventional DMARDs (Emery et al., 2008).

Like other biologicals, the higher prices of etanercept increase the financial burdens, biosimilars can bring cost savings for patients and emphasizes the necessity of patients access to therapies (Abraham et al., 2014; Simoens, 2021). Meanwhile, biosimilars for patients with RA are needed, particularly in countries with high levels of co-pay such as Central and Eastern European countries, due to concerns with their funding and utilization (Putrik et al., 2014; Yoo 2014; Baumgart et al., 2019). We have seen considerable reductions in the prices of biosimilars to help with their usage (Jensen et al., 2020; Moorkens et al., 2020), lower prices can also help increase the number of potential patients eligible for treatment within universal healthcare systems (Dutta et al., 2020). Despite this still see low use of biosimilars for anti-TNFs in some countries especially where limited price differences between the biosimilars and originators and limited demand-side measures encouraging the preferential prescribing of biosimilars (Kim et al., 2020; Tubic et al., 2021). This low uptake is exacerbated by concerns regarding immunogenicity leading to a heightened “Nocebo” effect and doubts about the efficacy and safety of biosimilars in practice (Colloca et al., 2019). The Norwegian Government-sponsored NOR SWITCH study on Infliximab was a good step in addressing the effectiveness and safety concerns associated with biosimilars, thus helping to enhance their use (Jørgensen et al., 2017), however, it is good to build upon this with studies and reviews like this one! Therefore, clarifying the differences in efficacy, safety, and immunogenicity of etanercept biosimilars *versus* reference biologics (Enbrel^®^) for RA by meta-analysis is necessary.

## 2 Materials and methods

This meta-analysis was mainly carried out and reported according to the guidelines of the Preferred Reporting Items for Systematic Review and Meta-analyses (PRISMA 2020) statement (Page et al., 2021). We register this meta-analysis on the International Prospective Register of Systematic Review (PROSPERO, CRD42022358709).

### 2.1 Literature search

With a combination of keywords and MeSH (Medical Subject Headings) terms related to rheumatoid arthritis, etanercept, and biosimilars, we searched PubMed, Embase (*via* ovid), Central (*via* ovid) comprehensively from inception to 15 August 2022 (Supplementary Table S2). We also searched ClinicalTrials.gov from database creation to August 15th for trials without published articles. In addition, reference lists of included literatures were screened to identify potential eligible studies.

### 2.2 Eligible criteria

The inclusion criteria were as follows: 1) Participants: adult patients diagnosed with rheumatoid arthritis who had an inadequate clinical response to methotrexate (MTX). 2) Interventions: an etanercept biosimilar with background MTX and folic acid. 3) Comparisons: an etanercept reference biologic (Enbrel^®^). 4) Outcomes: efficacy endpoints included proportion of patients achieving at least 20%, 50%, or 70% improvement in the American College of Rheumatology (ACR) response criteria (ACR20, ACR50, or ACR70) from per-protocol set (PPS) or full analysis set (FAS) at different time points; safety assessments included monitoring and recording of any adverse events, serious adverse events, withdrawal due to adverse events, and all-cause mortality; immunogenicity outcome was assessed using a bridging assay with anti-Hu-IgG detection of bound antidrug antibodies (ADAs). 5) Study design: randomized controlled trials (RCTs) in English language with follow up of at least 24 weeks.

Major exclusion criteria included 1) patients were previously treated with any other biologicals therapy for RA except etanercept within 3 months; 2) patients diagnosed with any active inflammatory or immune diseases except RA, congestive heart failure, active *tuberculosis*, or pregnancy.

### 2.3 Screening process and data extraction

After an initial screening of titles and abstracts, full-text articles were identified and reviewed in detail. As a quality check, two independent researchers (RH and SZ) screened separately titles and abstracts and assessed full-text articles, with a third researcher (TY) resolving any disagreements, if needed. For each selected article, the two researchers (RH and SZ) extracted the following data in an Excel spreadsheet: study characteristics (country, design, study period, and setting) and study population (case definition, eligible criteria, sample size, age, and sex).

### 2.4 Risk of bias assessment

In this study, we assessed risk of bias (RH and SZ) using the revised Cochrane Risk of Bias in Randomised Trials tool (RoB 2) (Sterne et al., 2019), which consists of five dimensions, bias arising due to: 1) the randomization process (D1); 2) deviations from intended interventions (D2); 3) missing outcome data (D3); 4) measurement of the outcome (D4); and 5) selection of the reported result (D5). After the first assessment, the tables were compared and disagreements were discussed.

### 2.5 Certainty of evidence assessment

The Grading of Recommendation Assessment, Development, and Evaluation (GRADE) framework was used to assess the certainty of the evidence according to risk of bias, inconsistency, imprecision, publication bias and indirectness (Guyatt et al., 2008; Zeng et al., 2021). Two authors assessed the certainty of evidence independently and rated to each comparison an overall qualitative judgment based on four levels of quality of evidence: high, moderate, low, very low; any disagreements were solved by discussions.

### 2.6 Statistical analysis

We performed two-stage meta-analysis pooling the Odds Ratio (OR) with their 95% Confidence Intervals (CIs) from extracted data to evaluate efficacy at different time points, safety, and immunogenicity of etanercept biosimilars *versus* etanercept reference biologics. Inconsistency test (*I* ^*2*^) was used to assess heterogeneity among included studies. The meta-analysis was calculated using the random effect model when *I* ^*2*^ was greater than 50%, which indicates a high probability of heterogeneity; as an alternative, the fixed-effects-based meta-analysis was conducted; Mantel-Haenszel method was used in our meta-analysis (Greenland and Robins, 1985; Robins et al., 1986; Borenstein et al., 2010; Bakbergenuly et al., 2020). We considered *p* values below 0.05 to be statistically significant.

We planned to address publication bias visually with funnel plots and statistically with the Egger regression when 10 or more studies were available. When fewer than 10 and at least five studies were included, we planned to use funnel plots and trim-and-fill analyses only (Duval and Tweedie, 2000).

We planned sensitivity analyses of studies with low risk of bias.

Our meta-analysis was conducted using the *meta* and *metafor* package in R software, version 4.2.1 (R foundation) (Viechtbauer, 2010; Polanin et al., 2017; Balduzzi et al., 2019).

## 3 Results

### 3.1 Study characteristics

**FIGURE 1:**
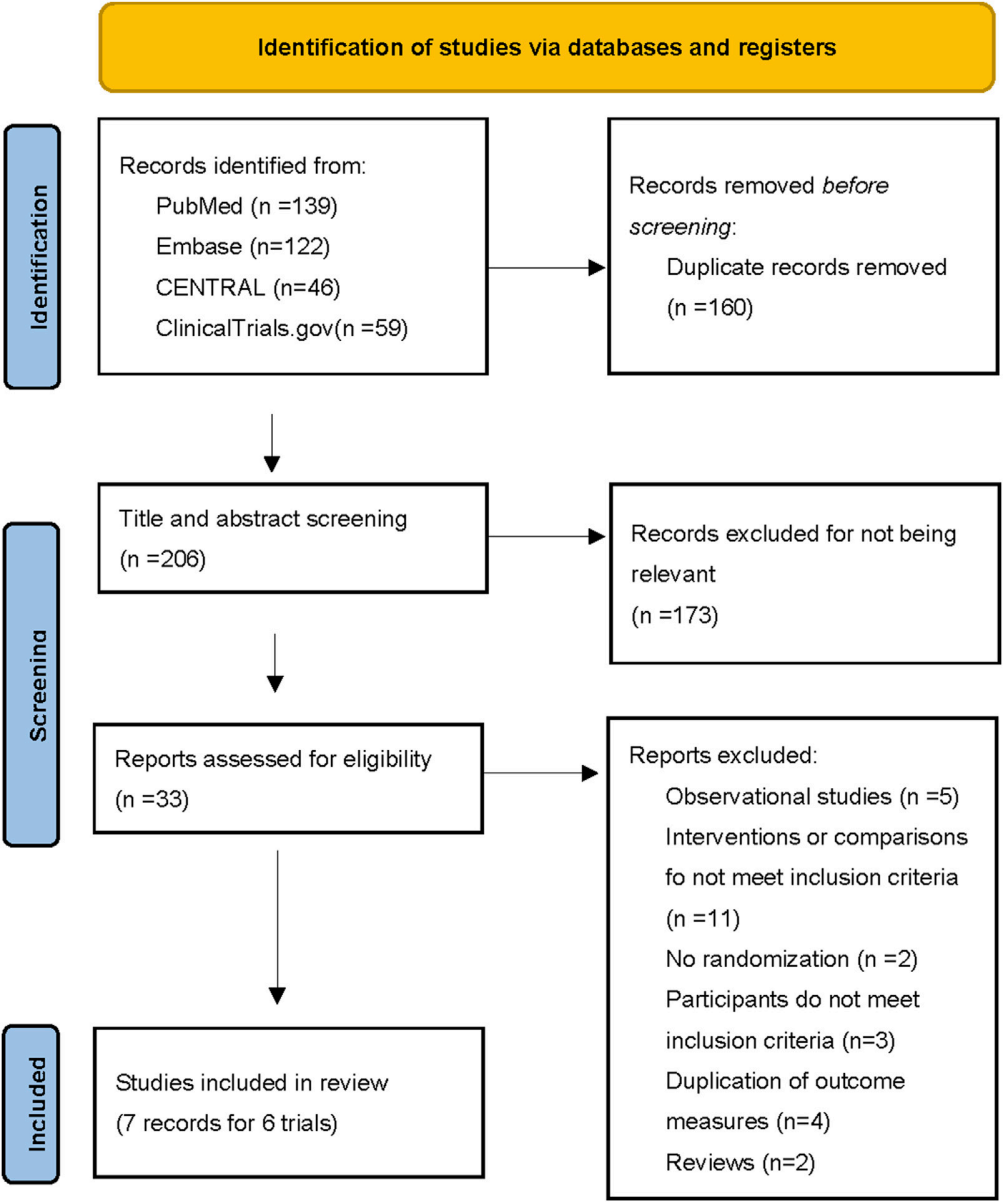
Flow diagram of literature search and selection.

A total of 366 records were identified from the databases, of which 206 unique records were screened after removing duplicate records. Out of these, the team assessed 33 full manuscripts for eligibility; 7 records from six RCTs with 2432 patients were included in our meta-analysis (Figure 1) (Bae et al., 2016; O'Dell et al., 2016; Emery et al., 2017a; Emery et al., 2017b; Matsuno et al., 2018; Matucci-Cerinic et al., 2018; Strusberg et al., 2021).

The baseline characteristics of the included studies were summarized in Table 1. The included trials registered were two-arm, conducted in various countries or regions (Korea, Europe, United States, Mexico, *Argentina*, Japan, South Africa), all of which were published in English. The mean age of participants ranged from 46 to 55 years old, the proportion of females ranged from 79.4% to 88.8%, the disease duration of patients ranged from 6 to 10 years, and the length of follow up of ranged from 24 to 52 weeks.

**TABLE 1:** Baseline characteristics of included studies (*n* = 6).

Register id	Biosimilar	Participants number		Age		Female		BMI		Disease duration	
		I	C	I	C	I	C	I	C	I	C
NCT01270997	HD203	147	146	51.0 ± 12.0	51.3 ± 12.4	101 (87.8%)	101 (85.6%)	22.5 ± 3.4	22.8 ± 3.5	7.19 ± 7.39	8.05 ± 7.43
NCT01895309	SB4(Benepali)	299	297	52.1 ± 11.72	51.6 ± 11.63	249 (83.3%)	253 (85.2%)	26.8 ± 5.51	26.3 ± 5.3	6.0 ± 4.2	6.2 ± 4.41
NCT02638259	GP2015(Erelzi)	186	190	55.2 ± 11.22	53.1 ± 12.7	158 (84.9%)	150 (78.9%)	\		8.79 ± 8.25	8.18 ± 6.92
NCT03332719	EtaBS(Enerceptan)	99	50	50.2 ± 10.8	46.3 ± 11.6	85 (85.9%)	42 (84%)	28.4 ± 5.1	27.9 ± 4.9	10.6 ± 8.8	10.5 ± 7
NCT02357069	LBEC0101	187	187	52.8 ± 11.6	55.5 ± 10.9	150 (81.1%)	166 (88.8%)	—		7.6 ± 7.6	7.8 ± 7.6
NCT02115750	CHS-0214	324	320	—		260 (80.2%)	254 (79.4%)	—		—	

I, interventions, etanercept biosimilars; C, comparisons, etanercept originators (Enbrel^®^); BMI, body mass index.

### 3.2 Risk of bias in eligible trials

**FIGURE 2:**
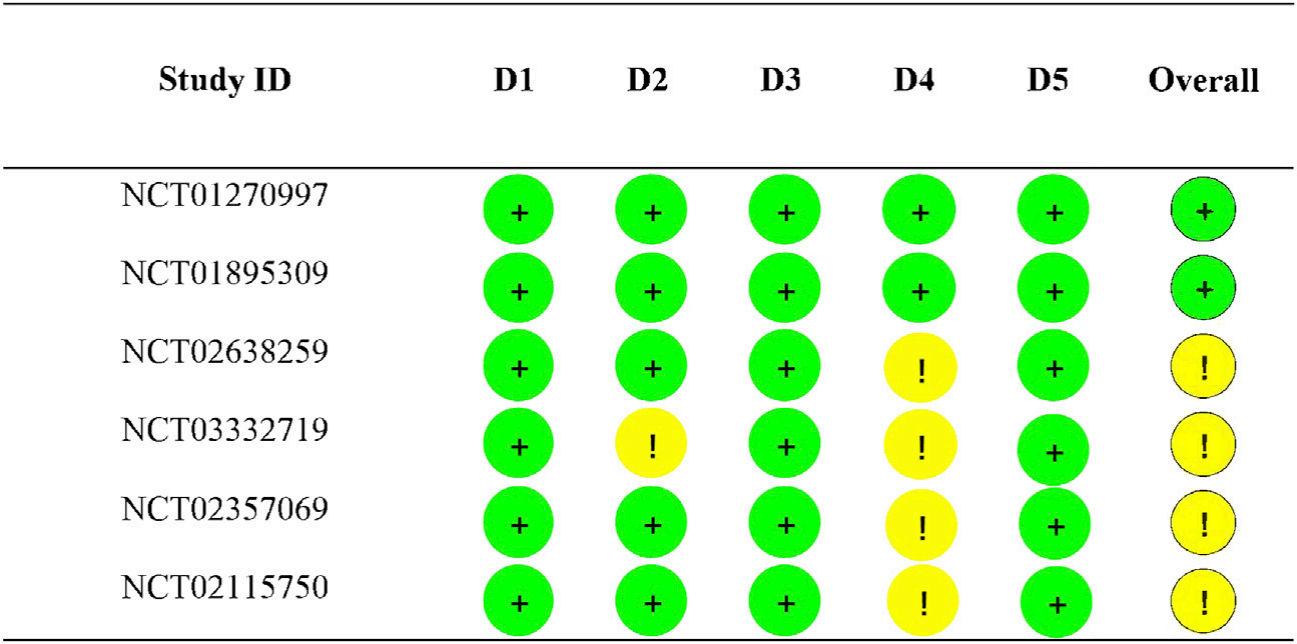
Risk of bias assessment results. D1: Randomization process; D2: Deviations from the intended interventions; D3: Missing outcome data; D4: Measurement of the outcome; D5: Selection of the reported results; + represents low risk; ! represents some concerns.

The assessment for risk of bias about included RCTs are presented in Figure 2. Specifically, NCT03332719 was at some concern for risk of bias because of evaluator-blinded and early termination; three trials (NCT02638259, NCT02357069, and NCT02115750) were at some concern for risk of bias because of early termination; other two trials were evaluated at low risk of bias in all domain.

### 3.3 Results of meta-analysis

#### 3.3.1 Efficacy

ACR20, ACR50, or ACR70 from PPS at different time points: the pooled results showed no significant difference in ACR20 at 24 weeks [5 RCTs, OR = 0.92 (0.54, 1.58), *p* = 0.79, *I* ^*2*^ = 73%, low certainty] or 1 year [3 RCTs, OR = 1.08 (0.76, 1.55), *p* = 0.66, *I* ^*2*^ = 0%, moderate certainty], and ACR70 at 24 weeks [5 RCTs, OR = 1.06 (0.87, 1.28), *p* = 0.58, *I* ^*2*^ = 45%, moderate certainty] between biosimilars and reference biologics (Enbrel^®^); biosimilars showed more benefits in ACR50 at 24 weeks [5 RCTs, OR = 1.22 (1.01, 1.47), *p* = 0.04, *I* ^*2*^ = 49%, high certainty] or 1 year [3 RCTs, OR = 1.43 (1.10, 1.86), *p* < 0.01, *I* ^*2*^ = 0%, high certainty], and ACR70 at 1 year [3 RCTs, OR = 1.32 (1.01, 1.71), *p* = 0.04, *I* ^*2*^ = 0%, high certainty] than reference biologics.

ACR20, ACR50, or ACR70 from FAS at different time points: the pooled results showed no significant difference in ACR20 at 24 weeks [2 RCTs, OR = 1.14 (0.84, 1.55), *p* = 0.39, *I* ^*2*^ = 0%, moderate certainty] or 32 weeks [2 RCTs, OR = 1.02 (0.72, 1.45), *p* = 0.92, *I* ^*2*^ = 0%, moderate certainty] or 1 year [2 RCTs, OR = 1.22 (0.91, 1.65), *p* = 0.19, *I* ^*2*^ = 0%, moderate certainty], ACR50 at 24 weeks [2RCTs, OR = 1.31 (1.00, 1.71), *p* = 0.053, *I* ^*2*^ = 21%, moderate certainty], and ACR70 at 24 weeks [2 RCTs, OR = 1.14 (0.83, 1.56), *p* = 0.42, *I* ^*2*^ = 0%, moderate certainty] or 1 year [2 RCTs, OR = 1.25 (0.93, 1.68), *p* = 0.13, *I* ^*2*^ = 0%, moderate certainty] between biosimilars and reference biologics; biosimilars showed more benefits in ACR50 at 1 year [2 RCTs, OR = 1.36 (1.04, 1.78), *p* = 0.03, *I* ^*2*^ = 0%, high certainty] than reference biologics.

**FIGURE 3:**
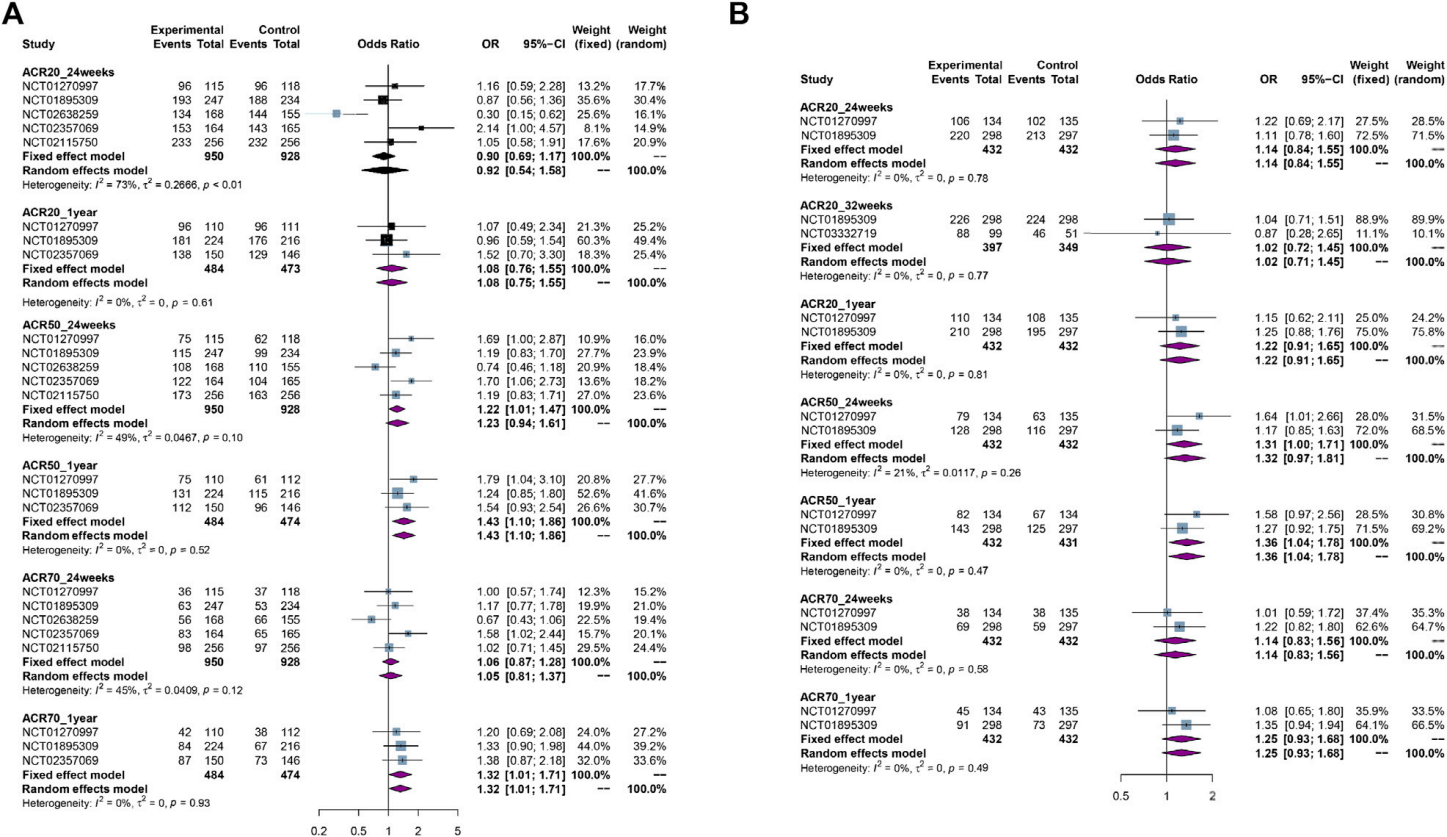
The forest plots of efficacy assessment. **(A)** ACR20, ACR50, ACR70 response rate from per-protocol set (PPS) at different time points. **(B)** ACR20, ACR50, ACR70 response rate from per-protocol set (PPS) at different time points.

The forest plots of efficacy assessment are presented in Figure 3.

#### 3.3.2 Safety

**FIGURE 4:**
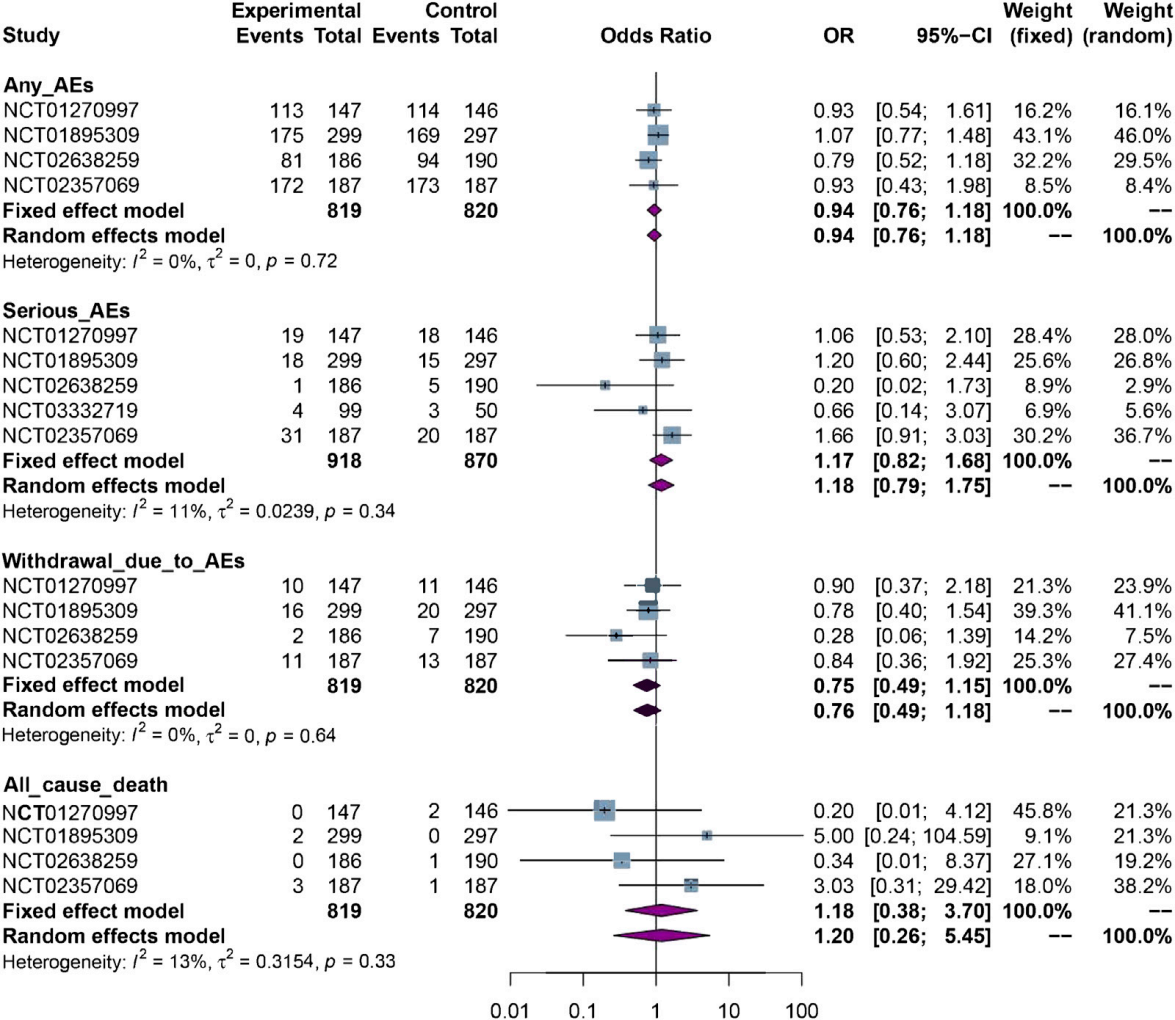
The forest plots of safety assessment. AEs: adverse events.

The results of meta-analysis showed there was no significant difference in incidence of any adverse events [4 RCTs, OR = 0.94 (0.76, 1.18), *p* = 0.61, *I* ^*2*^ = 0%, moderate certainty], incidence of serious adverse events [5 RCTs, OR = 1.17 (0.82, 1.68), *p* = 0.39, *I* ^*2*^ = 11%, moderate certainty], incidence of withdrawal due to adverse events [4 RCTs, OR = 0.75 (0.49, 1.15), *p* = 0.19, *I* ^*2*^ = 0%, moderate certainty], and all-cause mortality [4 RCTs, OR = 1.18 (0.38, 3.70), *p* = 0.77, *I* ^*2*^ = 13%, low certainty] between biosimilars and reference biologics. The forest plots of safety assessment are presented in Figure 4.

#### 3.3.3 Immunogenicity

**FIGURE 5:**
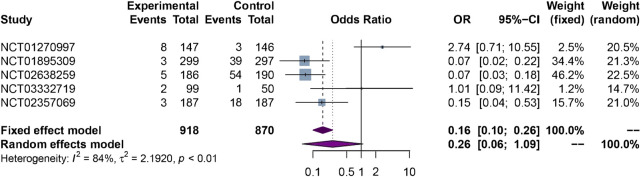
The forest plot of immunogenicity assessment.

The results of meta-analysis showed there was no significant difference in immunogenicity between biosimilars and reference biologics [5 RCTs, OR = 0.26 (0.06, 1.09), *p* = 0.07, *I* ^*2*^ = 84%, low certainty]. The forest plot of immunogenicity assessment are presented in Figure 5.

#### 3.3.4 Sensitivity analyses

The results of sensitivity analyses are presented in supplementary materials (Supplementary Section S3), and the robustness of our meta-analysis was confirmed by all sensitivity analyses.

### 3.4 Publication bias

Due to the limited number of included studies, we did not assess the publication bias by Egger regression. Funnel plots were performed to qualitatively evaluate the potential publication bias for outcomes involving at least five studies and trim-and-fill analyses were conducted to quantitatively assess the robustness. The assessment of publication bias is presented in supplementary materials (Supplementary Section S4). The results confirmed the robustness of primary findings.

## 4 Discussion

Our meta-analysis provides a summary of evidence regarding the efficacy, safety, and immunogenicity between an etanercept biosimilar and a reference biologic (Enbrel^®^) in the treatment of patients with RA. Our study suggests etanercept biosimilars are beneficial for RA patients with an inadequate response to MTX, with highly strong evidences showing ACR50 improvements at 24 weeks from PPS and at 1 year from both PPS and FAS, as well as ACR70 improvement at 1 year from PPS; and moderate and low evidence presented in the supplementary materials. In combination with the results of the evaluation of efficacy from PPS or FAS above, we hold the opinion that etanercept biosimilars may show more benefits in ACR50 at 1 year than reference biologics, and are similar to the reference biologic in terms of other outcomes including efficacy, safety and immunogenicity.

Although all six of the RCTs included had etanercept as a reference biologic in their control groups, biosimilars in each study was different, which may result in heterogeneity among the included studies. However, the results of sensitivity analyses and assessments of publication bias confirmed the robustness of our main results, so the heterogeneity in our meta-analysis was acceptable to us.

Before our study, there was a network meta-analysis which only included three etanercept biosimilars from three RCTs, indicated that no significant differences was found between etanercept biosimilars and etanercept originators in patients with RA despite treatment with MTX in terms of ACR20 response rate and incidence of serious adverse events, which was consistent with our findings (Lee and Song, 2021). Furthermore, some systematic literature reviews or network meta-analyses evaluated the efficacy and safety of tumor necrosis factor (TNF) inhibitors biosimilars *versus* TNF inhibitors originators, demonstrated that these biosimilars had an overall comparable efficacy and safety profile compared with their originators in RA patients, which was also similar to what we found (Komaki et al., 2017; Moots et al., 2018; Graudal et al., 2019; Ho Lee and Gyu Song, 2021). Nevertheless, theses previous related studies have some weakness, such as limited outcome indicators, failure to consider different time points and a lack of evidence assessment.

To the best of our knowledge, our meta-analysis is the first meta-analysis with the focus on efficacy, safety, and immunogenicity including as many outcome indicators in different time points as possible for RA patients. In addition, we used sensitivity analyses to confirm the robustness of our main results and trim-and-fill analyses to address the publication bias. Our meta-analysis is not only an update of the data of relevant meta-analysis or network meta-analysis before, but also provides an overview of the evidence for comparisons between etanercept biosimilars and reference biologics.

There are several limitations to our study. First, we only included phase III RCTs which had strict eligible criteria and may not truly reflect real-world conditions due to the differences between large randomized controlled trials and real-world practice (Zhou et al., 2021). At present there are some studies evaluated etanercept biosimilars *versus* etanercept originators in RA patients based on real-world data. And all these studies demonstrated the biosimilars were as effective, safe, and acceptable as the original (Codreanu et al., 2019; Atzeni et al., 2021; Gharibdoost et al., 2021; Rojas-Giménez et al., 2021; Selmi et al., 2021; Pinto et al., 2022). The conclusions of other real-world studies are similar to ours; we considered the efficiency outcomes at different time points and found that biosimilars had relative benefits in some efficacy outcomes at those times. Therefore, our study provides a reference for clinical practice and decision making, and offers preliminary evidence to support future observational studies. We would not discuss any superiority of the biosimilar compared to the originator; rather, we would just state that they are as good. This may be difficult for key personnel to appreciate, especially if they initially harbored doubts about biosimilars. Differences in efficacy may be attributed to the fact that originator manufacturers often change their manufacturing processes, which could have an effect on effectiveness. However, it is difficult to definitively state this since originator manufacturers do not need to conduct new studies following changes in manufacturing processes (Vezér et al., 2016; Jiménez-Pichardo et al., 2017; Godman et al., 2020). Now the published studies address the lack of problems for patients switching between biosimilars, which is the next big hurdle to address in order to further accelerate biosimilar use (Allocati et al., 2022).

The conclusions of these real-world studies are somewhat similar to ours, but we considered the efficiency outcomes at different time points and we found biosimilars showed relative benefits in some efficacy outcomes at different times. Thus, our study still provides reference for clinical practice and decision making, and preliminary evidence to support future observational studies. Second, as RA is a chronic disease, it would be preferable to conduct time-to-event outcome analysis, but relevant data are not available, so we can only evaluate outcomes at different time points by two-stage meta-analysis. Third, we did not conduct Egger regression due to the limited number of included studies, the risk of publication bias could not be excluded by funnel plots, but the trim-and-fill analysis confirmed the robustness of our results.

## 5 Conclusion

Etanercept biosimilars showed more benefits in ACR50 response rate at 1 year than reference biologics, other outcomes for clinical efficacy, safety, and immunogenicity of etanercept biosimilars were comparable with originator in RA patients with background MTX and folic acid. The main results of our study further support the utilization of etanercept biosimilars in clinical practice and decision making. And more future studies based on real-world data are needed to validate the findings of our study.

## Data Availability

The original contributions presented in the study are included in the article/Supplementary Material, further inquiries can be directed to the corresponding authors.
