# Supplementary material for: Efficacy, safety and immunogenicity of etanercept biosimilars versus reference biologics in patients with rheumatoid arthritis: A meta-analysis
Source: Front Pharmacol. 2023 Feb 16;14:1089272. doi: 10.3389/fphar.2023.1089272 (PMC9979087; doi:10.3389/fphar.2023.1089272)
Supplement: Supplementary file 1 [file DataSheet1.pdf]

Supplementary materials for Efficacy, Safety and Immunogenicity of Etanercept Biosimilars versus Reference Biologics in Patients with Rheumatoid Arthritis: A Meta-analysis

Contents

Section 1. PRISMA checklist **2**

Section 2. Search strategy **5**

Section 3. The results of sensitivity analyses **6**

Section 4. Assessment of publication bias **9**

Section 5. Results of GRADE assessment **14**

Section 1. PRISMA checklist

Table S1. PRISMA checklist

Section and Topic	Item #	Checklist item	Location where item is reported
**TITLE**			
Title	1	Identify the report as a systematic review.	Lines 1-3
**ABSTRACT**			
Abstract	2	See the PRISMA 2020 for Abstracts checklist.	Lines 17-40
**INTRODUCTION**			
Rationale	3	Describe the rationale for the review in the context of existing knowledge.	Lines 43-63
Objectives	4	Provide an explicit statement of the objective(s) or question(s) the review addresses.	Lines 63-66
**METHODS**			
Eligibility criteria	5	Specify the inclusion and exclusion criteria for the review and how studies were grouped for the syntheses.	Lines 80-95
Information sources	6	Specify all databases, registers, websites, organisations, reference lists and other sources searched or consulted to identify studies. Specify the date when each source was last searched or consulted.	Lines 73-78
Search strategy	7	Present the full search strategies for all databases, registers and websites, including any filters and limits used.	Section 2 in supplementary materials
Selection process	8	Specify the methods used to decide whether a study met the inclusion criteria of the review, including how many reviewers screened each record and each report retrieved, whether they worked independently, and if applicable, details of automation tools used in the process.	Lines 97-100
Data collection process	9	Specify the methods used to collect data from reports, including how many reviewers collected data from each report, whether they worked independently, any processes for obtaining or confirming data from study investigators, and if applicable, details of automation tools used in the process.	Lines 100-103
Data items	10a	List and define all outcomes for which data were sought. Specify whether all results that were compatible with each outcome domain in each study were sought (e.g. for all measures, time points, analyses), and if not, the methods used to decide which results to collect.	Lines 100-103
	10b	List and define all other variables for which data were sought (e.g. participant and intervention characteristics, funding sources). Describe any assumptions made about any missing or unclear information.	Lines 100-103
Study risk of bias assessment	11	Specify the methods used to assess risk of bias in the included studies, including details of the tool(s) used, how many reviewers assessed each study and whether they worked independently, and if applicable, details of automation tools used in the process.	Lines 105-110
Effect measures	12	Specify for each outcome the effect measure(s) (e.g. risk ratio, mean difference) used in the synthesis or presentation of results.	Lines 120-121
Synthesis methods	13a	Describe the processes used to decide which studies were eligible for each synthesis (e.g. tabulating the study intervention characteristics and comparing against the planned groups for each synthesis (item #5)).	Lines 83-89

Section and Topic	Item #	Checklist item	Location where item is reported
	13b	Describe any methods required to prepare the data for presentation or synthesis, such as handling of missing summary statistics, or data conversions.	Lines 121
	13c	Describe any methods used to tabulate or visually display results of individual studies and syntheses.	Lines 124-129
	13d	Describe any methods used to synthesize results and provide a rationale for the choice(s). If meta-analysis was performed, describe the model(s), method(s) to identify the presence and extent of statistical heterogeneity, and software package(s) used.	Lines 124-128
	13e	Describe any methods used to explore possible causes of heterogeneity among study results (e.g. subgroup analysis, meta-regression).	Lines 123-124
	13f	Describe any sensitivity analyses conducted to assess robustness of the synthesized results.	Lines 134
Reporting bias assessment	14	Describe any methods used to assess risk of bias due to missing results in a synthesis (arising from reporting biases).	Lines 105-110
Certainty assessment	15	Describe any methods used to assess certainty (or confidence) in the body of evidence for an outcome.	Lines 112-118
**RESULTS**			
Study selection	16a	Describe the results of the search and selection process, from the number of records identified in the search to the number of studies included in the review, ideally using a flow diagram.	Figure 1, lines140-144
	16b	Cite studies that might appear to meet the inclusion criteria, but which were excluded, and explain why they were excluded.	Figure 1
Study characteristics	17	Cite each included study and present its characteristics.	Table 1
Risk of bias in studies	18	Present assessments of risk of bias for each included study.	Figure 2, lines 153-157
Results of individual studies	19	For all outcomes, present, for each study: (a) summary statistics for each group (where appropriate) and (b) an effect estimate and its precision (e.g. confidence/credible interval), ideally using structured tables or plots.	Figure 3, 4, 5
Results of syntheses	20a	For each synthesis, briefly summarise the characteristics and risk of bias among contributing studies.	Figure 3, 4, 5; lines 160-192
	20b	Present results of all statistical syntheses conducted. If meta-analysis was done, present for each the summary estimate and its precision (e.g. confidence/credible interval) and measures of statistical heterogeneity. If comparing groups, describe the direction of the effect.	Figure 3, 4, 5; lines 160-192
	20c	Present results of all investigations of possible causes of heterogeneity among study results.	Lines 215-217
	20d	Present results of all sensitivity analyses conducted to assess the robustness of the synthesized results.	Section 3 in supplementary materials
Reporting biases	21	Present assessments of risk of bias due to missing results (arising from reporting biases) for each synthesis assessed.	Section 4 in supplementary materials
Certainty of evidence	22	Present assessments of certainty (or confidence) in the body of evidence for each outcome assessed.	Section 5 in supplementary materials

Section and Topic	Item #	Checklist item	Location where item is reported
**DISCUSSION**			
Discussion	23a	Provide a general interpretation of the results in the context of other evidence.	Lines 204-214
	23b	Discuss any limitations of the evidence included in the review.	Lines 240-256
	23c	Discuss any limitations of the review processes used.	Lines 240-256
	23d	Discuss implications of the results for practice, policy, and future research.	Lines 250-251
**OTHER INFORMATION**			
Registration and protocol	24a	Provide registration information for the review, including register name and registration number, or state that the review was not registered.	Lines 70-71
	24b	Indicate where the review protocol can be accessed, or state that a protocol was not prepared.	
	24c	Describe and explain any amendments to information provided at registration or in the protocol.	
Support	25	Describe sources of financial or non-financial support for the review, and the role of the funders or sponsors in the review.	Lines 276-278
Competing interests	26	Declare any competing interests of review authors.	Lines 280-281
Availability of data, code and other materials	27	Report which of the following are publicly available and where they can be found: template data collection forms; data extracted from included studies; data used for all analyses; analytic code; any other materials used in the review.	Lines 265-267

Section 2. Search strategy

Table S2. Peer-reviewed literature search strategy.

**PubMed search terms**	((("Arthritis, Rheumatoid"[Mesh]) OR (Rheumatoid arthritis[Title/Abstract])) AND (("Etanercept"[Mesh]) OR (etanercept[Title/Abstract]))) AND (("Biosimilar Pharmaceuticals"[Mesh] OR "etanercept biosimilar SB4" [Supplementary Concept]) OR (biosimilar[Title/Abstract])) AND ("Randomized Controlled Trials as Topic"[Mesh] OR "Randomized Controlled Trial" [Publication Type] OR "randomized controlled trials")
**Embase search terms**	' Rheumatoid arthritis'/mp OR ' rheumatoid arthritis '/ AND ([etanercept]/mp OR [etanercept]/ AND [biosimilar]/mp OR [biosimilar agent]/) AND [randomized controlled trial]/mp OR [randomized controlled trial]/)
**Central search terms**	' Rheumatoid arthritis'/mp OR ' rheumatoid, arthritis '/ AND ([etanercept]/mp OR [etanercept]/ AND [biosimilar]/mp OR [biosimilar pharmaceuticals]/) AND [randomized controlled trial]/mp OR [randomized controlled trial]/)

Section 3. The results of sensitivity analyses

3.1 ACR20, ACR50, ACR70 response rate from per-protocol set

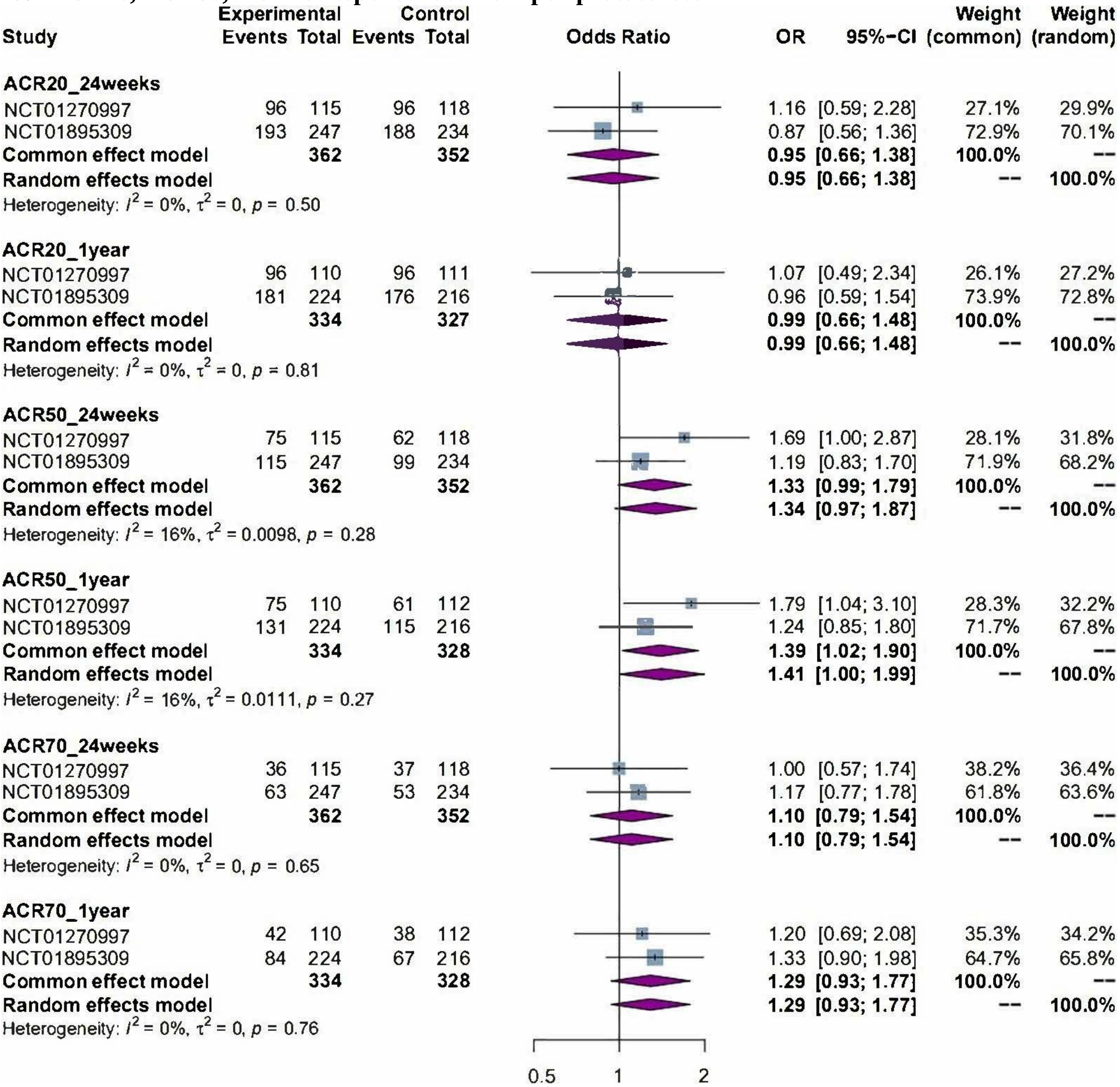

3.2 ACR20, ACR50, ACR70 response rate from full-analysis set

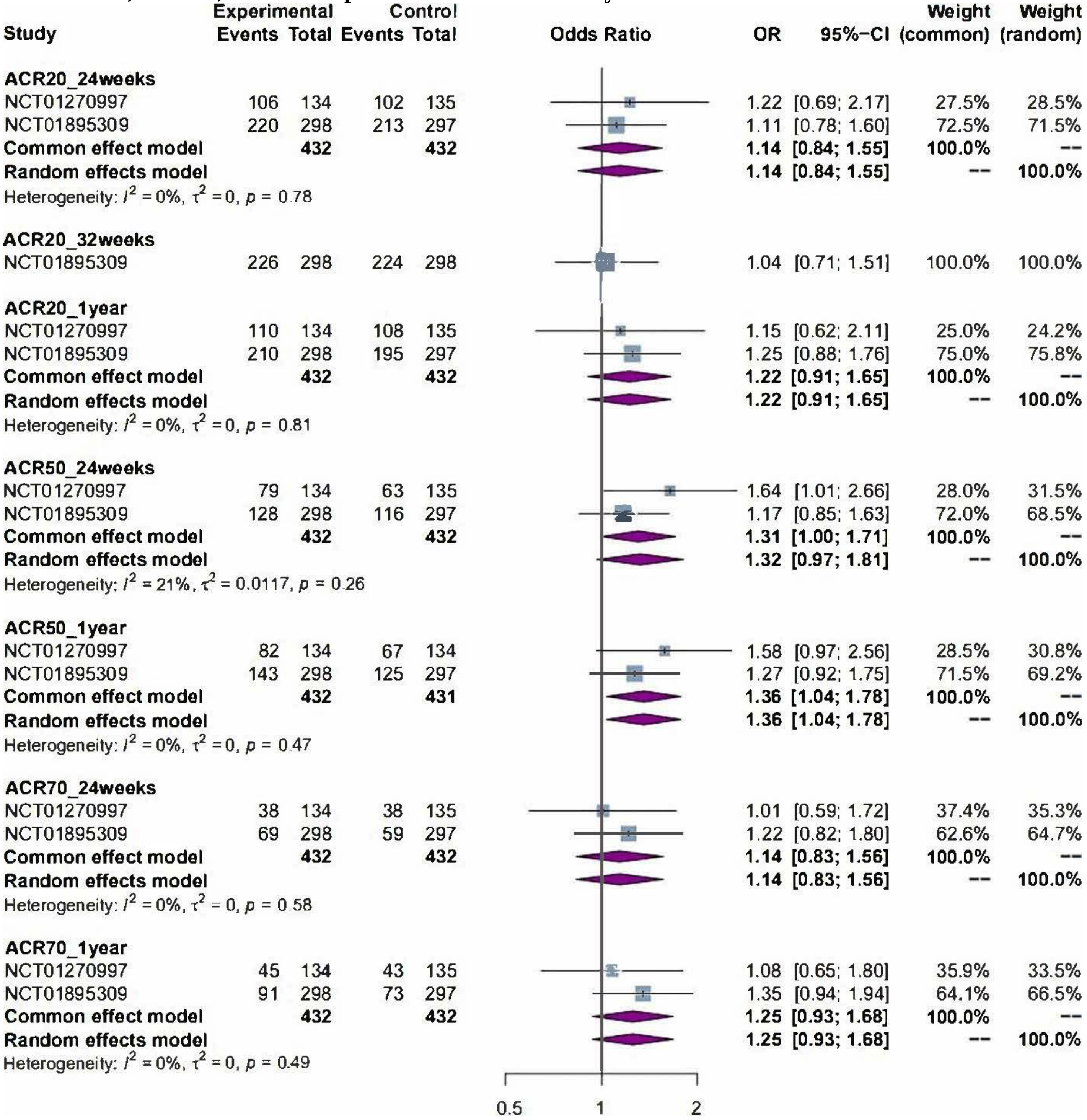

3.3 Safety assessments

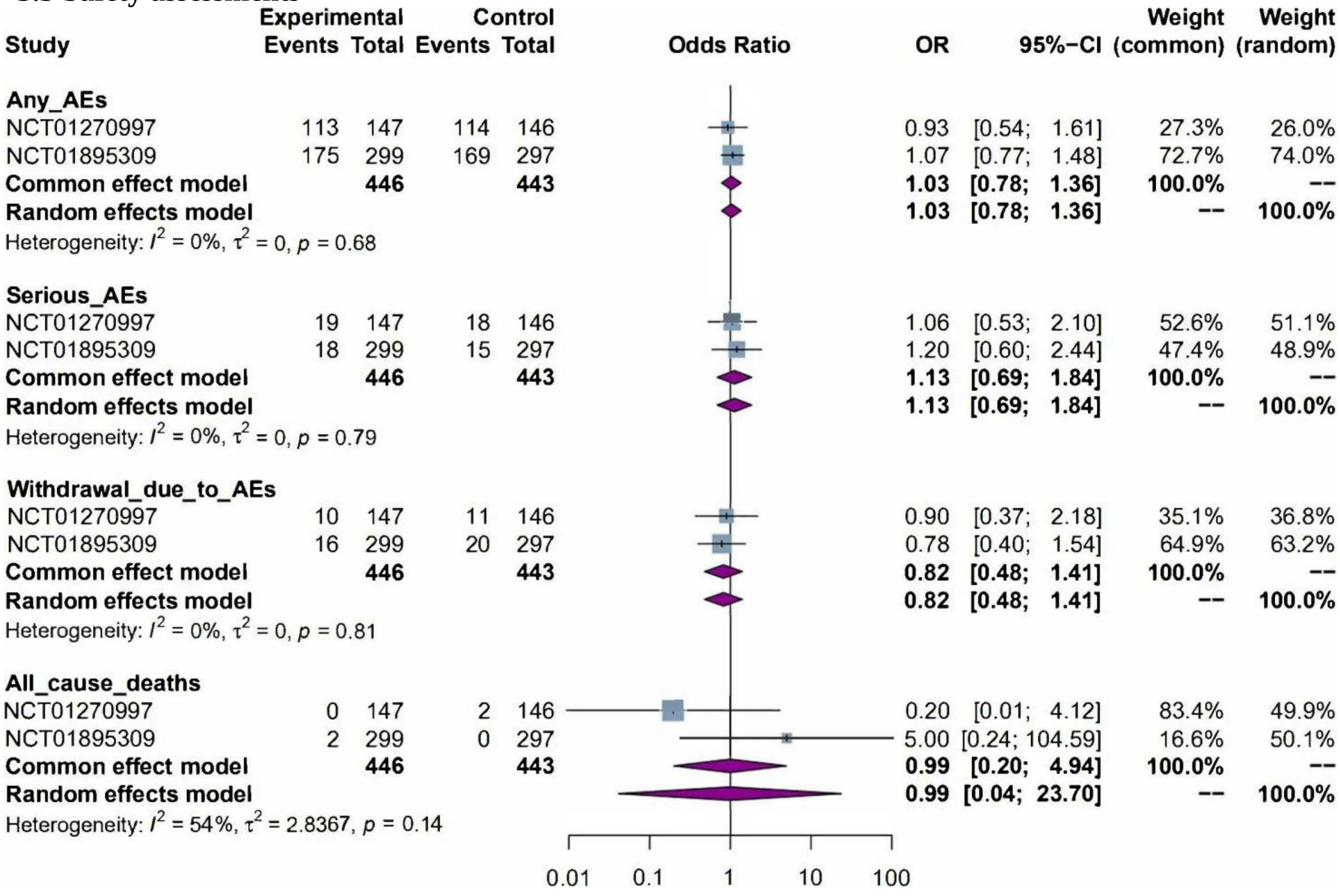

3.3 Immunogenicity assessments

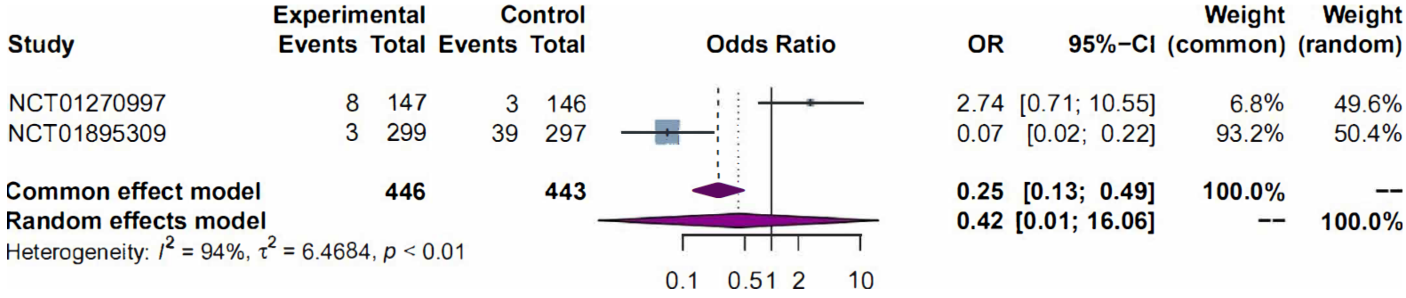

Section 4. Assessment of publication bias

4.1 ACR20 response rate at 24 weeks

4.1.1 Original funnel plots

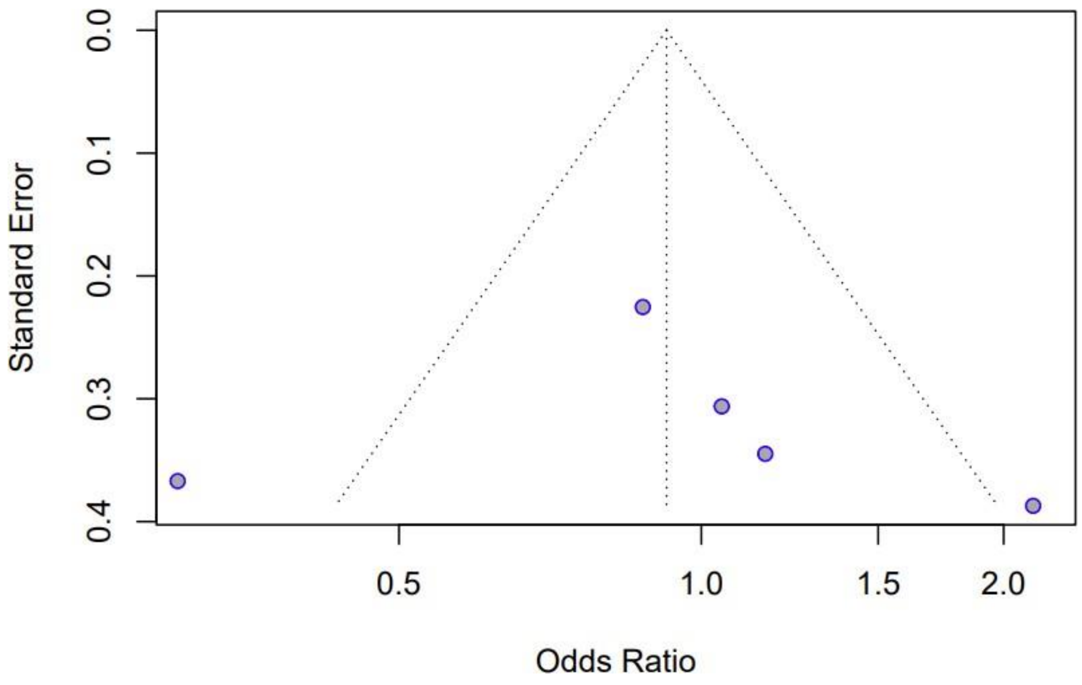

4.1.2 Trim-and-fill funnel plots

Note: Adjusted odds ratio with one filled study was 0.77 with 95% confidence interval 0.42 to 1.41.

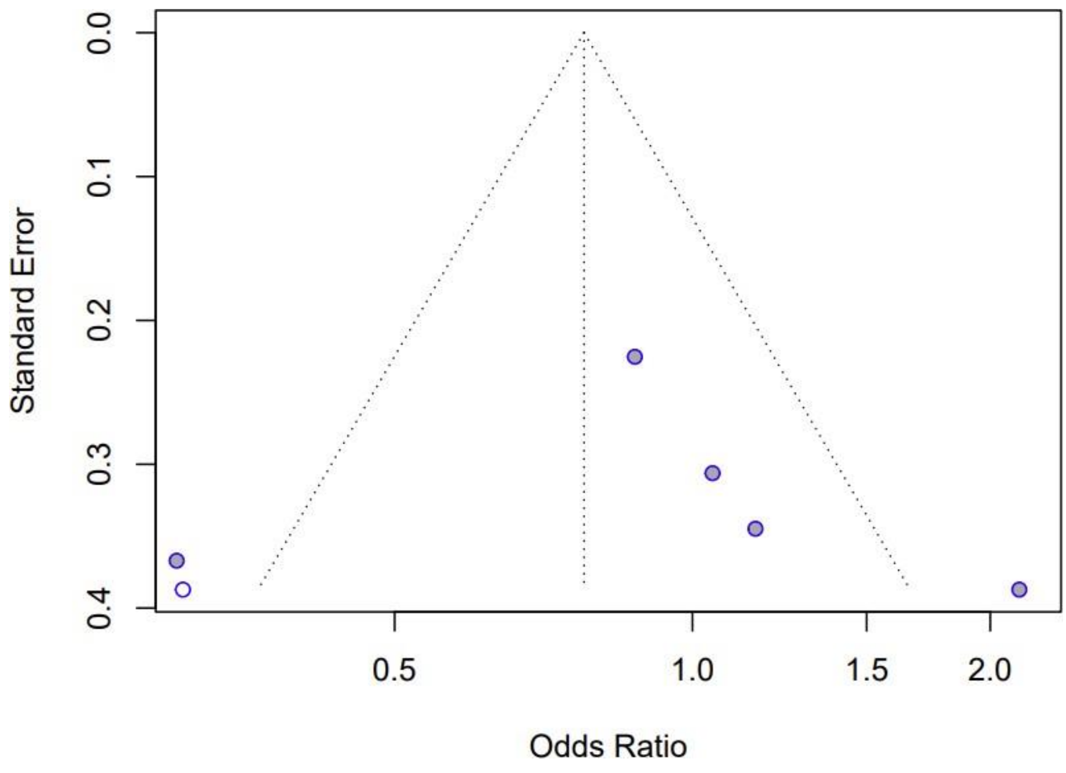

4.2 ACR50 response rate at 24 weeks

4.2.1 Original funnel plots

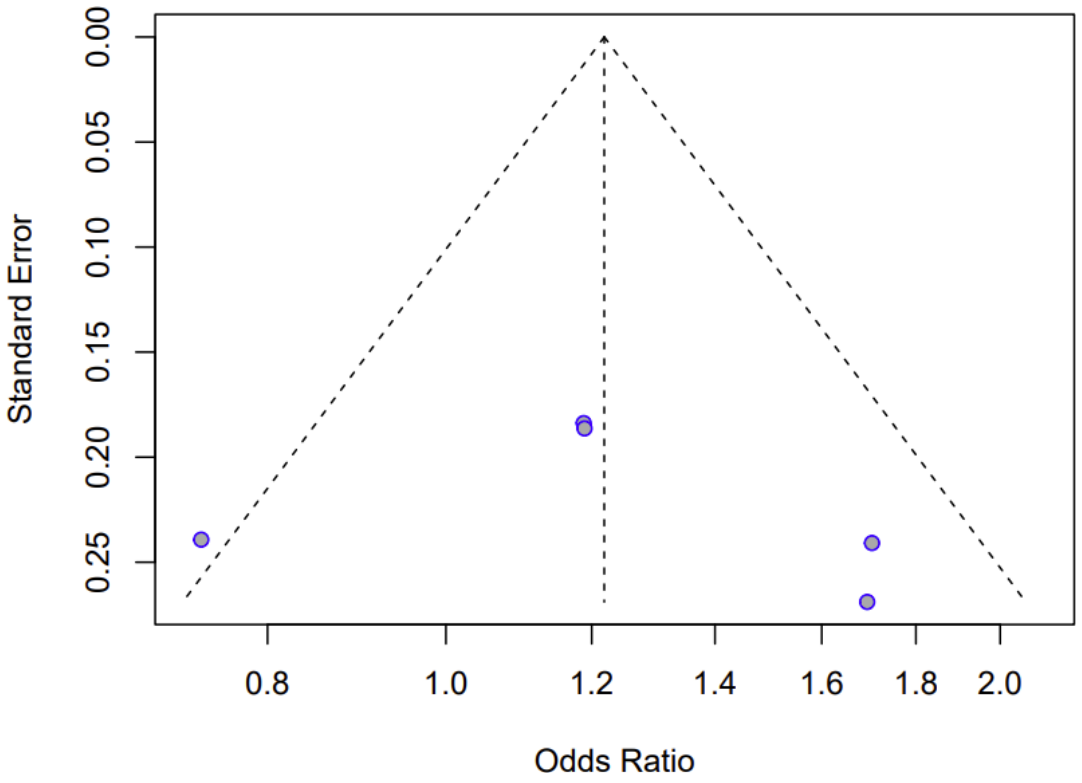

4.2.2 Trim-and-fill funnel plots

Note: No study was filled.

4.3 ACR70 response rate at 24 weeks

4.3.1 Original funnel plots

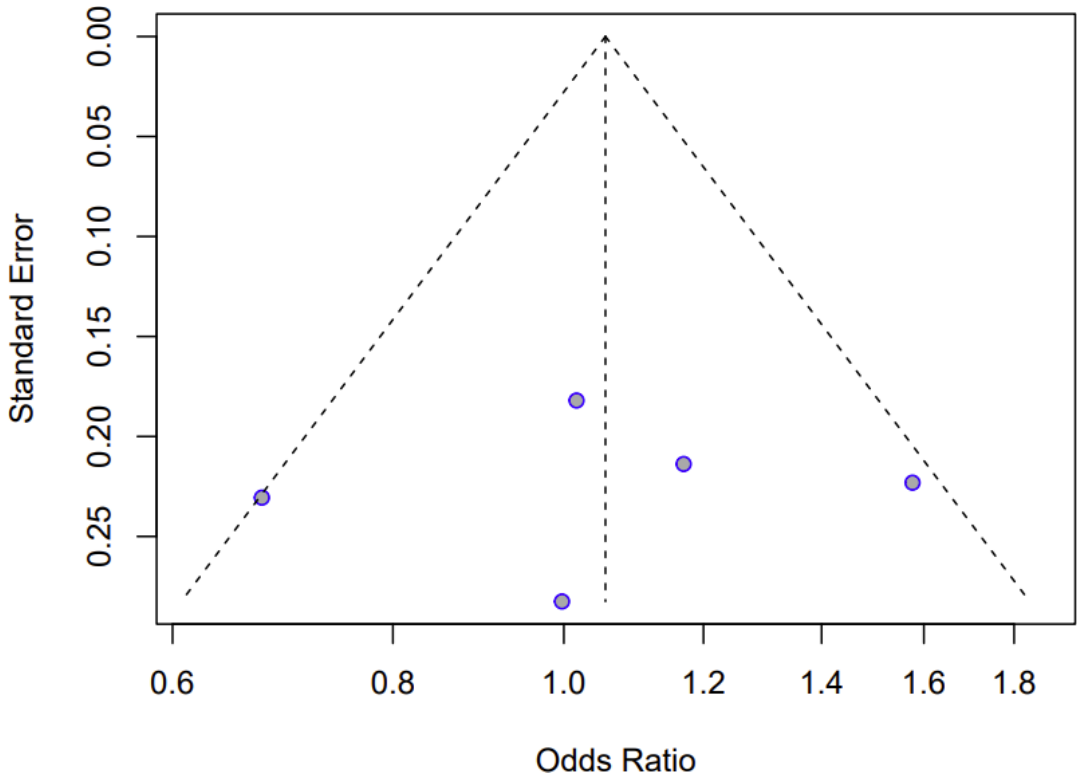

4.3.2 Trim-and-fill funnel plots

Note: No study was filled.

4.4 Incidence of serious adverse events

4.4.1 Original funnel plots

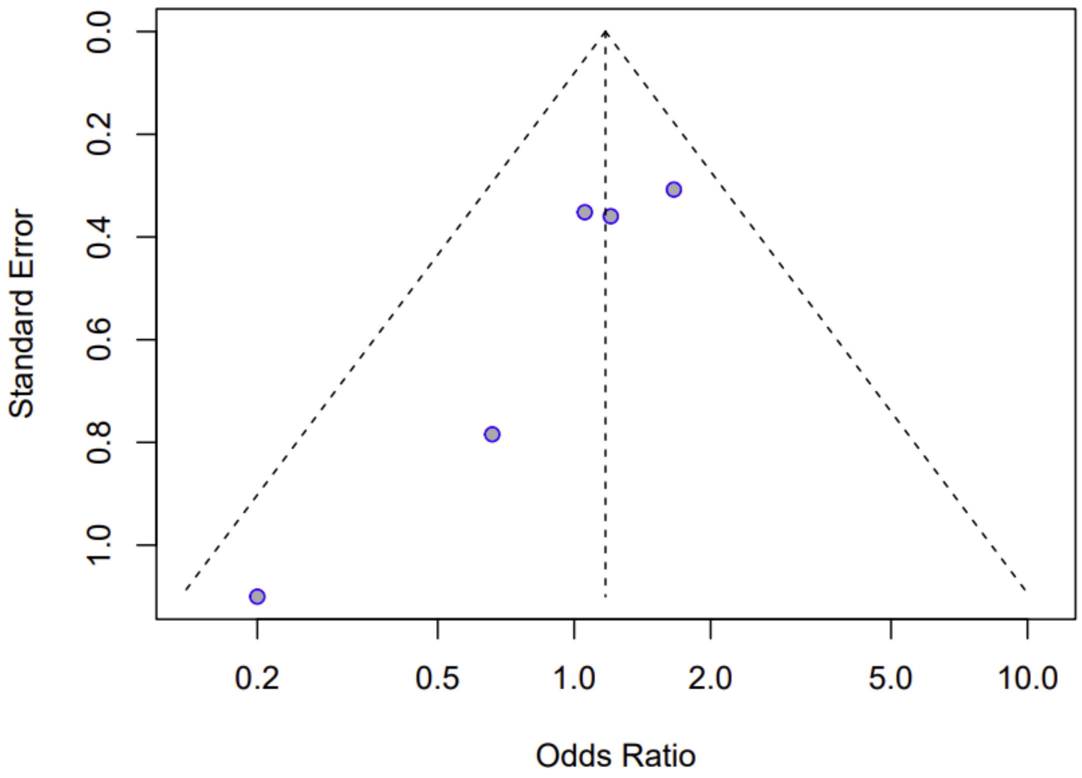

4.4.2 Trim-and-fill funnel plots

Note: Adjusted odds ratio with two filled study was 1.32 with 95% confidence interval 0.93 to 1.87.

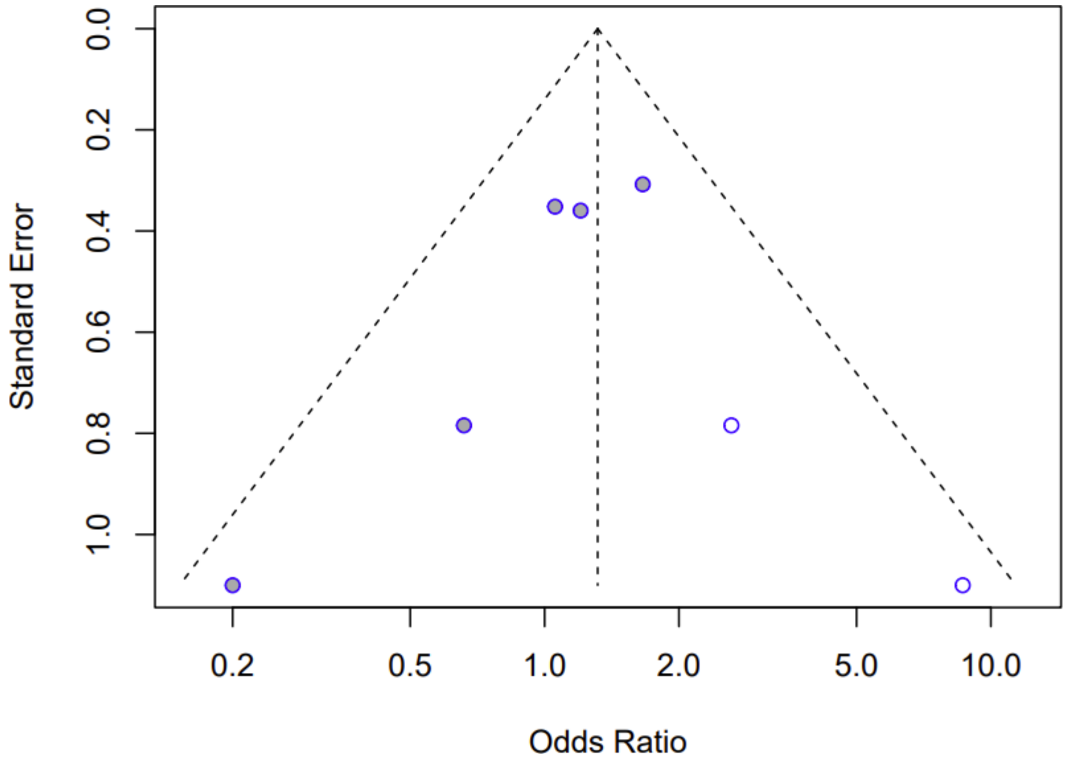

4.5 Assessment of immunogenicity

4.5.1 Original funnel plots

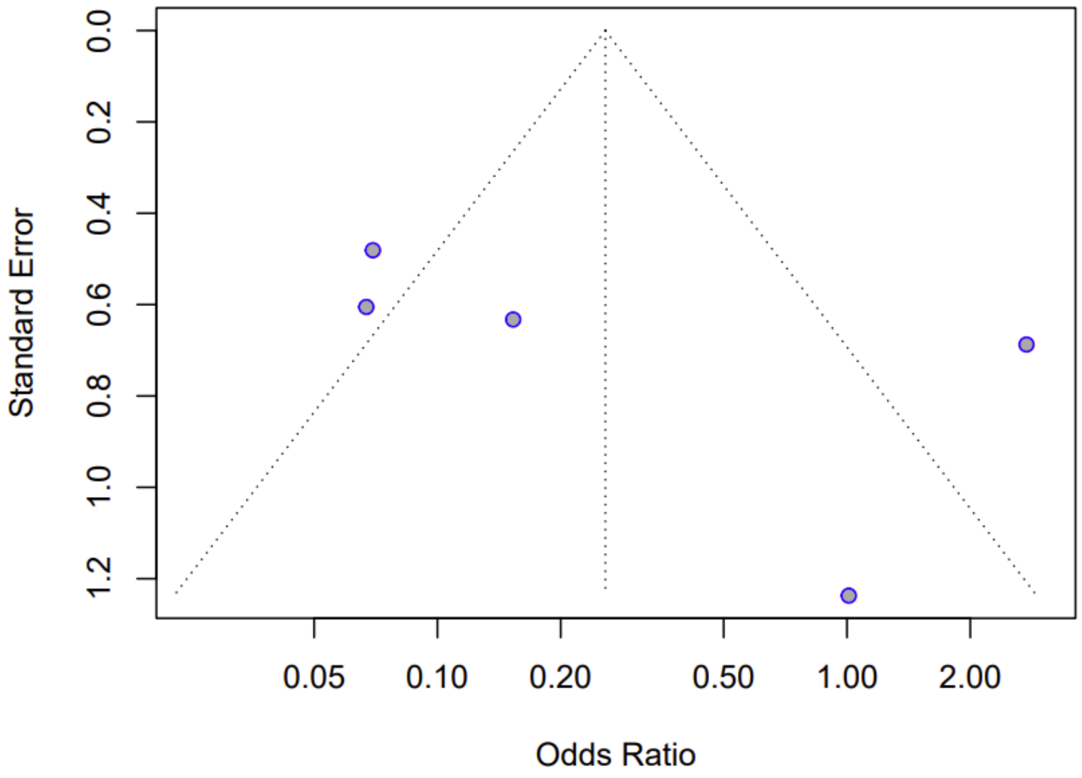

4.5.2 Trim-and-fill funnel plots

Note: Adjusted odds ratio with two filled study was 0.09 with 95% confidence interval 0.01 to 0.53.

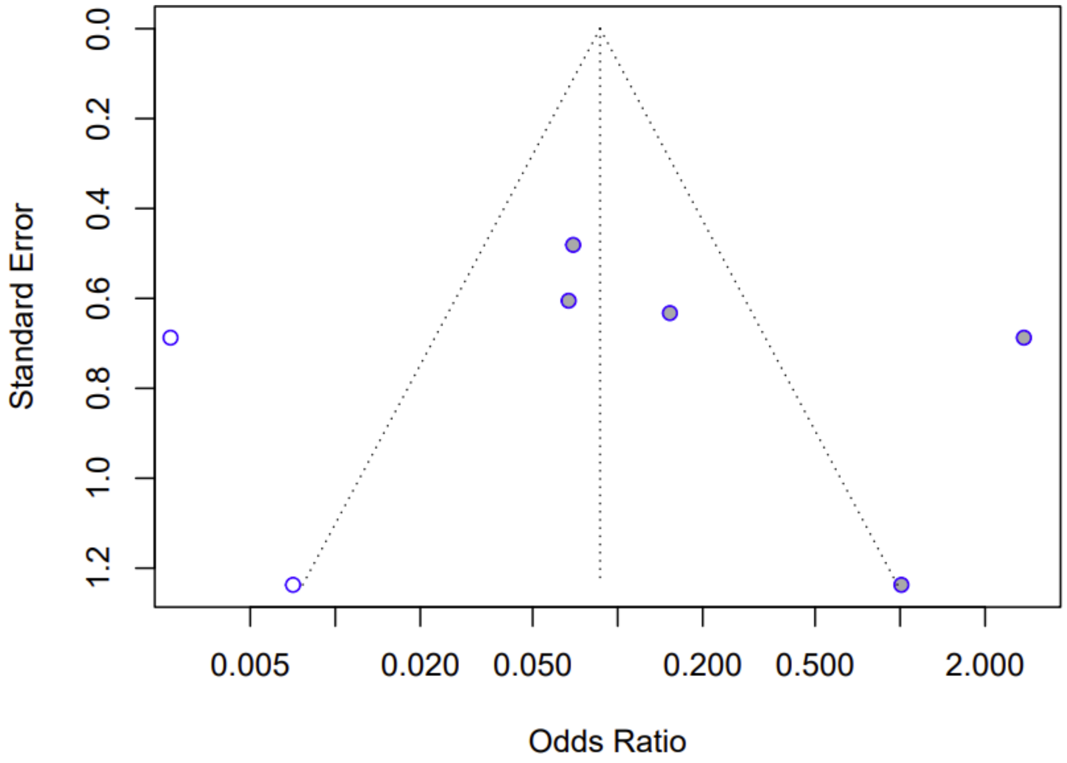

Section 5. Results of GRADE assessment

biosimiars compared to etanercept for RA

Patient or population: patients with RA
Settings:
Intervention: biosimiars
Comparison: etanercept

Outcomes	Illustrative comparative risks* (95% CI)<br>Assumed risk<br>Etanercept	 Corresponding risk<br>Biosimiars	Relative effect (95% CI)	No of Participants (studies)	Quality of the evidence (GRADE)	Comments
ACR20_24weeks_PPS	**Study population**		**OR 0.92** (0.54 to 1.58)	1878 (5 studies)	⊕⊕⊝⊝ **low**[1,2]	
	**865 per 1000**	**855 per 1000** (776 to 910)				
	**Moderate**					
ACR20_1year_PPS	**Study population**		**OR 1.08** (0.76 to 1.55)	957 (3 studies)	⊕⊕⊕⊝ **moderate**[2]	
	**848 per 1000**	**857 per 1000** (809 to 896)				
	**Moderate**					
ACR50_24weeks_PPS	**Study population**		**OR 1.22** (1.01 to 1.47)	1878 (5 studies)	⊕⊕⊕⊕ **high**	
	**580 per 1000**	**627 per 1000** (582 to 670)				
	**Moderate**					
ACR50_1year_PPS	**Study population**		**OR 1.43** (1.10 to 1.86)	958 (3 studies)	⊕⊕⊕⊕ **high**	
	**574 per 1000**	**658 per 1000** (597 to 715)				
	**Moderate**					
ACR70_24weeks_PPS	**Study population**		**OR 1.06** (0.87 to 1.28)	1878 (5 studies)	⊕⊕⊕⊝ **moderate**[2]	
	**343 per 1000**	**356 per 1000** (312 to 400)				
	**Moderate**					
ACR70_1year_PPS	**Study population**		**OR 1.32** (1.01 to 1.71)	958 (3 studies)	⊕⊕⊕⊕ **high**	
	**376 per 1000**	**443 per 1000** (378 to 507)				
	**Moderate**					
ACR20_24weeks_FAS	**Study population**		**OR 1.14** (0.84 to 1.55)	864 (2 studies)	⊕⊕⊕⊝ **moderate**[2]	
	**729 per 1000**	**754 per 1000** (693 to 807)				
	**Moderate**					
ACR20_32weeks_FAS	**Study population**		**OR 1.02** (0.72 to 1.45)	746 (2 studies)	⊕⊕⊕⊝ **moderate**[2]	
	**774 per 1000**	**777 per 1000** (711 to 832)				
	**Moderate**					
ACR20_1year_FAS	**Study population**		**OR 1.22** (0.91 to 1.65)	864 (2 studies)	⊕⊕⊕⊝ **moderate**[2]	
	**701 per 1000**	**741 per 1000** (681 to 795)				
	**Moderate**					
ACR50_24weeks_FAS	**Study population**		**OR 1.31** (1.00 to 1.71)	864 (2 studies)	⊕⊕⊕⊝ **moderate**[2]	
	**414 per 1000**	**481 per 1000** (414 to 547)				
	**Moderate**					
ACR50_1year_FAS	**Study population**		**OR 1.36** (1.04 to 1.78)	863 (2 studies)	⊕⊕⊕⊕ **high**	
	**445 per 1000**	**522 per 1000** (455 to 588)				
	**Moderate**					
ACR70_24weeks_FAS	**Study population**		**OR 1.14** (0.83 to 1.56)	864 (2 studies)	⊕⊕⊕⊝ **moderate**[2]	
	**225 per 1000**	**248 per 1000** (194 to 311)				
	**Moderate**					
ACR70_1year_FAS	**Study population**		**OR 1.25** (0.93 to 1.68)	864 (2 studies)	⊕⊕⊕⊝ **moderate**[2]	
	**269 per 1000**	**315 per 1000** (255 to 381)				
	**Moderate**					
Any_AEs	**Study population**		**OR 0.94** (0.76 to 1.18)	1639 (4 studies)	⊕⊕⊕⊝ **moderate**[2]	
	**671 per 1000**	**657 per 1000** (608 to 706)				
	**Moderate**					
Serious_AEs	**Study population**		**OR 1.17** (0.82 to 1.68)	1788 (5 studies)	⊕⊕⊕⊝ **moderate**[2]	
	**70 per 1000**	**81 per 1000** (58 to 112)				
	**Moderate**					
Withdrawal_due_to_AEs	**Study population**		**OR 0.75** (0.49 to 1.15)	1639 (4 studies)	⊕⊕⊕⊝ **moderate**[2]	
	**62 per 1000**	**47 per 1000** (31 to 71)				
	**Moderate**					
All_cause_deaths	**Study population**		**OR 1.18** (0.38 to 3.70)	1639 (4 studies)	⊕⊕⊝⊝ **low**[3]	
	**5 per 1000**	**6 per 1000** (2 to 18)				
	**Moderate**					
Immunogenicity	**Study population**		**OR 0.26** (0.06 to 1.09)	1788 (5 studies)	⊕⊕⊝⊝ **low**[1,3,4]	
	**132 per 1000**	**38 per 1000** (9 to 142)				
	**Moderate**					

*The basis for the **assumed risk** (e.g. the median control group risk across studies) is provided in footnotes. The **corresponding risk** (and its 95% confidence interval) is based on the assumed risk in the comparison group and the **relative effect** of the intervention (and its 95% CI).

CI: Confidence interval; **OR:** Odds ratio;

GRADE Working Group grades of evidence
High quality: Further research is very unlikely to change our confidence in the estimate of effect.
Moderate quality: Further research is likely to have an important impact on our confidence in the estimate of effect and may change the estimate.
Low quality: Further research is very likely to have an important impact on our confidence in the estimate of effect and is likely to change the estimate.
Very low quality: We are very uncertain about the estimate.

1 hetergeneity
2 imprecision
3 serious imprecision
4 large effect
